# Russian Lepers

**Published:** 1892-08

**Authors:** 


### RUSSIAN LEPERS.

Miss Kate Marsden is to visit Canada and the United States. She has just returned from Siberia, from away up the Lena river. She is now in St. Petersburg, and has told her story of her long journey of thousands of miles through Siberia, where she has been hunting for lepers. She says she has found a few settlements of these outcasts, and now she intends to lecture for the purpose of securing funds to provide medical care for the unfortunates and to enable her to go and place them in colonies, and so to win glory, as did Father Damien in Molakai, in the Sandwich Islands.

From the story she tells, she has apparently been to Viliusk, a place about 200 miles north of Yakootsk, on the Lena river. There, she says, she found lepers banished to the forests, where they were kept away from the rest of the people, but fed by the latter on fish and tree-bark. Thirty guides, she says, were obliged to cut out a path through the undergrowth of the forests in order that she could reach the leper villages. She found the stricken people ill clad, living in indescribable degradation, and many of them so loathsome in appearance as to have lost all semblance to humanity.

Miss Marsden further says that there has been found in Yakootsk a plant that is a sure cure for leprosy, but that she has not been able to test it as yet. A year ago, she met near Samara the Bishop Dionysius of the Ufa, who had labored at Yakootsk for a period of over forty years, and he had seen the plant and had known of cures of leprosy accomplished by its agency.

The lepers live in a settlement apart, on the banks of the Vilui river. Here a number of huts have been erected for their accommodation and here they live in an indescribable condition of filth and immorality. In spite, however, of such unfavorable conditions, by the use of the plant cures are said to be effected. The plant is found growing wild on the banks of a small river called the Ugur. The discovery of its curative virtues was due to a curious accident. It appears that at this place a leper had been living with his family. His state at length became such that they could no longer suffer him to dwell in the house, and he was accordingly turned out into the fields. His relatives each day brought him a certain quantity of food, but, as may readily be imagined his strength rapidly declined, till at length he could no longer walk, and was forced all day to lie prostrate on the ground.

After a few days he was surprised to observe that his sores commenced to heal, and it was finally discovered that this was owing to the contact with a certain plant which grew there in abundance Further experiments were made, and it was found that the application of this plant to the leprous sores caused them to heal. Unfortunately there is no proper medical aid in the district, so that until now no scientific observation of its effects has been made. Bishop Dionysius informed Miss Marsden that a young doctor had gone to live among the lepers, with a view of investigating scientifically the action of the plant, but he unfortunately took ill and died before he had time to make any communication on the subject. One peculiar discovery he made during his stay, and that was the discovery of the leper bacillus in a fish common in the district. He attributed to the eating of this fish the prevalence of leprosy in and around Yakutsk.

Miss Marsden made her journey under the most favorable auspices. She was provided with an autograph letter from the Czarina, with whom she had been in constant communication. She made the journey from Moscow as far as Omsk in company with a young Englishwoman named Miss Field. In one of her letters from the north, Miss Marsden wrote: "Dr. Alexeeff and myself are now by the banks of the Lena waiting till our cargo boat starts. We have found out that we must ride on horseback from Yakutsk to Viluisk, a thousand versts, then ride about finding the lepers, which may mean another 2,000, then 1,000 versts back again to Yakutsk. I have to ride all these thousands of versts, as there are no roads, only marshes and forests, and I rather dread it, I must confess. To begin with, I have been obliged to have trousers made and very high boots over the knees, as I must ride like a man; they say it is too unsafe to ride the other way, as we have constantly to get off the horses and wade through either the water or forests, so I shall be a little tired when I get back to England, but I would go through fire and water to help my poor lepers."

Miss Marsden's most remarkable feat is doubtless her long journey in summer and winter. Her talk about lepers will, presumably, be interesting, and her idea of caring for the poor creatures is a humanitarian one. But there is no cure for leprosy on the Lena. Russian and Polish doctors have studied the disease thoroughly. It was taken there in its primitive form a hundred years ago by exiles. The disease, an infectious one, has, in many cases, died out in the villages where it was originally planted, in the course of generations. But occasionally children are born with the hideous hereditary disease on them, which develops to what is styled leprosy, but is quite incurable. The poor creatures seem to bear upon themselves the collective inherited punishment of past generations.

